# Cardiorespiratory Fitness and Health Outcomes Across the Spectra of Age, Gender, and Race

**DOI:** 10.31083/j.rcm2501015

**Published:** 2024-01-10

**Authors:** Peter Kokkinos, Jonathan Myers

**Affiliations:** ^1^Department of Kinesiology and Health, School of Arts and Sciences, Rutgers University, New Brunswick, NJ 08901, USA; ^2^Veterans Affairs Medical Center, Washington, D.C. 20422, USA; ^3^George Washington University School of Medicine and Health Sciences, Washington, D.C. 20052, USA; ^4^Veterans Affairs Palo Alto Health Care System, Palo Alto, CA 94304, USA; ^5^Department of Cardiology, Stanford University, Stanford, CA 94305, USA

For the past seven decades, numerous studies have evaluated the association between physical activity (PA) status and health outcomes in diverse populations using self-reported occupational or recreational patterns of weekly PA to define and quantify fitness. In general, their findings supported the concept that higher PA has a favorable impact on human health [1, 2, 3, 4]. However, the quantification of fitness was solely based on highly subjective self-reported accounts, presenting a significant weakness of these studies. The paradigm shifted when Professor Steven Blair in his studies from the Cooper Clinic assessed cardiorespiratory fitness (CRF) objectively using a standardized exercise treadmill test (ETT) and stratified the cohort according to peak metabolic equivalents achieved (METs; 1 MET = 3.5 mL per kg of body weight per minute) [5, 6]. The evidence accumulated from a plethora of studies that followed confirmed overwhelmingly that the association between CRF and all-cause mortality was inverse, graded, and independent of known cardiovascular risk factors and comorbidities. This association was similar for both males and females and across the age, and race spectra [7, 8, 9, 10]. The definition of CRF according to peak METs achieved made it possible to quantify a change in risk of a specific health outcome (mortality or incidence of disease) per MET change in CRF and to establish MET threshold levels beyond which exercise-related health benefits are realized [8]. The change in risk has been reported to be between 10%–25% per each 1-MET increase in exercise capacity, depending on the population studied and disease burden [11, 12].

Studies further provided strong evidence of an independent and graded association between CRF, chronic disease incidence and the progression of chronic disease. Specific chronic diseases have included hypertension [13, 14] the progression rate from elevated blood pressure to hypertension [15, 16], incidence of type 2 diabetes mellitus (T2DM) [17, 18, 19] and the progression rate to insulin in patients diagnosed with T2DM, [18] heart failure [20, 21], major adverse cardiovascular events [22], chronic kidney disease [23, 24], and dyslipidemia [25, 26].

Despite this significant evolution in our quest to define the CRF-health outcomes association, the challenge to better understand the independent or synergistic effects of CRF and genetic factors on the CRF-health outcomes association persisted. Findings from relatively small studies indicated that improvements in CRF assessed objectively by sequential standardized ETTs are also associated with favorable health outcomes in mostly healthy populations, suggesting an independent effect of CRF apart from genetic factors [5]. The findings of a recent study with the largest cohort of its kind (n = 93,060) strengthened this concept [27]. Changes in CRF over time reflected inverse and proportional changes in mortality risk. These findings also expanded our understanding of the magnitude of CRF change necessary to alter mortality risk. Specifically, an increase in CRF of approximately ≥1.0 MET from the baseline evaluation was associated with a progressively lower mortality risk regardless of CRF status at baseline. Similarly, a decrease in CRF by ≥1.0 MET from baseline was associated with a progressive increase in mortality risk except in those with very high baseline CRF and no cardiovascular disease (CVD). Since the time between the two CRF assessments was at least 1 year with a mean of 5.8 ± 3.7 years, this suggests that CRF-related protection against premature mortality may be long-lasting when CRF status is relatively high [27].

These findings compel us to the following syllogism. If the health outcomes were determined solely by genetic factors, then changes in CRF would be less likely coincide with changes in mortality risk. However, the findings of the aforementioned study support the concept that changes in mortality risk were concomitant and proportional to changes in CRF regardless of initial CRF status. A more likely hypothesis is that participation in aerobic activities leads to structural and functional physiological adaptations and these adaptations render an individual more resistant to injury or disease, ultimately resulting in lower mortality independent of genetic factors. This makes a persuasive argument that the impact CRF is a determinant of all-cause mortality risk that is independent of genetic factors as suggested by others [28].

Although the CRF-health outcomes association follows a similar pattern across the age, gender, and race spectra, subtle but important differences have been noted in at least some studies, suggesting that the impact of CRF on health outcomes differs depending on the population studied. Thus, this volume is devoted to exploring potential differences in the CRF-health outcomes association across the race, gender, and age spectra. In addition, the interaction between PA, CRF, and non-traditional risk factors is explored, including chronic kidney disease, gut microbiota, sleep apnea, and atrial fibrillation. Finally, despite the wealth of evidence regarding the impact of PA on health, it remains underutilized as an intervention to reduce the incidence of chronic disease; thus, the implementation of PA and CRF as routine measures in clinical practice is discussed.
